# In-screw polymethylmethacrylate-augmented sacroiliac screw for the treatment of fragility fractures of the pelvis: a prospective, observational study with 1-year follow-up

**DOI:** 10.1186/s12893-017-0330-y

**Published:** 2017-12-08

**Authors:** Andreas Höch, Philipp Pieroh, Ralf Henkelmann, Christoph Josten, Jörg Böhme

**Affiliations:** 1 Department of Orthopedics, Trauma and Plastic Surgery, Spine Center, University Hospital of Leipzig, Liebigstraße 20, 04103 Leipzig, Germany; 2 Department of Anatomy and Cell Biology, Martin Luther University Halle-Wittenberg, Grosse Steinstrasse 52, 06097 Halle (Saale), Germany; 3 Present address: Clinic of Trauma, Orthopedic and Septic Surgery, Hospital St.Georg GmbH, Delitzscher Str. 141, 04129 Leipzig, Germany

**Keywords:** Fragility fracture, Pelvic fracture, Sacrum fracture, Polymethylmethacrylate, Sacroiliac screw fixation

## Abstract

**Background:**

The incidence of pelvic ring fractures in the elderly significantly increased. Because of persistent pain and immobilization associated with this injury, surgical treatment is recommended. To minimise comorbidities and surgical risk, percutaneous techniques are becoming more relevant. In-screw cement augmentation of sacroiliac screw fixation is a promising procedure; however, clinical follow-up data remain scarce. This study investigated the safety and possible complications of the procedure along with a 1-year follow-up.

**Methods:**

Thirty-four patients (treated with 43 screws) were prospectively included. Data on patients’ age and sex, the mechanism of accident, fracture pattern, duration of hospital stay, surgery and adverse events were recorded. Data were obtained postoperatively on the reduction of pain and complications, such as infection, cement leakage and neurological deficits, and at 1-year follow-up on pain, quality of life according to the 12-Item Short Form Survey and mobility. Implant failure was defined as retraction or dislocation of screws and was also documented.

**Results:**

Screw-related complications occurred with 2 of 43 screws. None of these complications were related to cement augmentation. In-hospital adverse events occurred in 6 of 34 patients. Postoperative pain, measured by the visual analogue scale, was significantly reduced from 6.7 ± 1.4 preoperatively to 2.7 ± 1.0 postoperatively (*p* < 0.001). Although patients complained of pain at the 1-year follow-up, they reported a significant decline compared with pain at admission (3.4 ± 2.3; p < 0.001). Results on the quality of life were comparable with those for the age- and gender matched German population. All patients were mobile, and no implant failure was detected.

**Conclusions:**

The results indicate that in-screw augmented sacroiliac screw fixation for fragility fractures of the pelvis is a safe technique. Pain was significantly reduced immediately after surgery compared to the preoperative state. Furthermore, significant pain reduction after one year compared to the preoperative state and quality of life was comparable to the age- and gender- matched German population. Thus, we recommend in-screw augmentation for screw fixation for sacral fragility fractures of the pelvis following failed conservative treatment.

## Background

The incidence of pelvic ring fractures in elderly patients has been increasing because of the ageing of the population [1–4]. In general, these fractures are associated with the combination of a low-energy trauma and an impaired bone stock [5, 6]. The predominant fracture morphology is a lateral compression fracture that involves the anterior pubic rami and an impression of the sacral massa lateralis [7]. Based on the increase of these fractures, Rommens and Hofmann established a new classification system to morphologically describe fragility fractures of the pelvis [8, 9]. Apart from causing pain and related immobilisation, lateral compression injuries have a tendency of becoming unstable during conservative treatment [3, 5, 10]. In addition to appropriate osteoporosis therapy and the use of painkillers, several authors recommend surgical treatment [2, 9, 11, 12].

Because of the high rate of comorbidities, postoperative infections and poor bone quality, percutaneous techniques combined with cement application have become more relevant [13–16]. In-screw cement augmentation with fenestrated screws yielded promising results in short-term follow-up examinations [14, 17]. However, long-term follow-up data remain lacking. The present study investigated pain, quality of life, mobility and implant failure in elderly patients who underwent in-screw polymethylmethacrylate (PMMA) augmented sacroiliac (SI) screw fixation at 1-year follow-up.

## Methods

### Ethical approval

The study was approved by the local ethics committee (297–12-24,092,012), and informed signed consent was obtained from all participating patients.

### Patients, study design and preoperative data acquisition

**Fig. 1:**
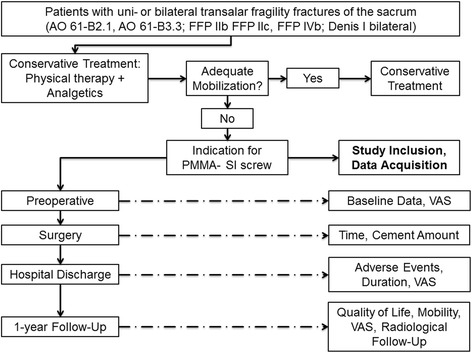
Study protocol, patient selection and data collection. All patients suffering from a uni- or bilateral transalar fagility fracture of the sacrum were treated primarily conservatively with physical therapy (full weight bearing) and painkillers in accordance to the World Health Organization (WHO) analgesic ladder step 2. If adequate mobilization was achieved, conservative treatment was continued. In cases of persistent immobilization the surgical treatment was indicated. Patients treated posteriorly solely by a PMMA augmented SI screw without additional posterior stabilization and independent of the anterior stabilization were asked preoperatively to participate in the present study. Data were collected at the indicated time points prospectively and reviewed retrospectively

The present study was carried out from July 2012 to December 2014. The study protocol is presented in Fig. 1. All patients suffering from a transalar fragility fracture of the sacrum classified as AO 61-B2.1, AO 61-B3.3, FFP IIb FFP IIc, FFP IVb or Denis I bilateral (Table 1) [7, 8] were evaluated. Initially all these patients were treated conservatively with physical therapy allowing full weight bearing. Painkillers were given according to the World Health Organization (WHO) analgesic ladder step 2. If mobilization was adequate, conservative treatment was continued (Fig.1). In cases of persistent immobilization surgical treatment was indicated. Patients from this patient population planned for in-screw PMMA augmented SI screw fixation independent of anterior fracture fixation, without additional posterior fixation (e.g. spinopelvic fixation) were asked for study participation and included in the study.

**Table 1:** Classification of included pelvic ring fractures according to OTA/AO and Rommens and Hofmann’s classification of fragility fractures of the pelvis (FFP) [8]

Classification	Fracture type	Number of patients
OTA/AO	B 2.1	25
	B 3.3	8
Isolated sacral fracture	Denis I bilateral	1
FFP	FFP 2b	11
	FFP 2c	14
	FFP 4b	8
	No classification possible	1

Thus, 34 patients (79 ± 8.25 years, 32 female and 2 male) were prospectively included. Nine patients sustained a bilateral Denis I fracture. Furthermore, in cases of unilateral sacral fractures, the corresponding anterior fracture was ipsilateral in 11 patients and contralateral in 10 patients. Four patients had a bilateral anterior fracture. Fracture classifications according to the Orthopaedic Trauma Association/Association for the Study of Internal Fixation (OTA/AO) and Rommens et al. are summarized in Table 1. Baseline data such as age, sex and type of admission, the mechanism of accident, injury pattern and comorbidities are presented in Table 2. Thirty-one patients had a history of minor trauma or no trauma. Eleven patients suffered from additional injuries (5 upper limb fractures, 3 osteoporotic spine fractures and 3 cerebral contusions). Moreover, preoperatively the pain level using the visual analogue scale (VAS) was recorded.

**Table 2:** Demographic data on age, sex, type of admission, comorbidities, mechanism of accident and injury pattern of the study population

	Number of patients
*N*	34
Age (years)	79 ± 8.25 (41–92)
Gender	32 females, 2 males; 16:1
Admission	13 primary admission
	21 secondary transfer
Comorbidities^a^	1 with no comorbidities
	24 with ≤ 3 comorbidities
	9 with > 3 comorbidities
Mechanism of accident	28 fell from a stand
	3 with unknown/no trauma
	1 because of a traffic accident
	2 fell from a height of > 3 m
Injury pattern	21 with isolated pelvic ring fracture
	10 with accompanying injuries (ISS < 16)
	3 with multiple injured (ISS > 16)

^a^hypertension, diabetes mellitus, cold, cardiac event in history, neurological event with residual

### Surgery- surgical technique, surgical time and amount of used bone cement

**Fig. 2:**
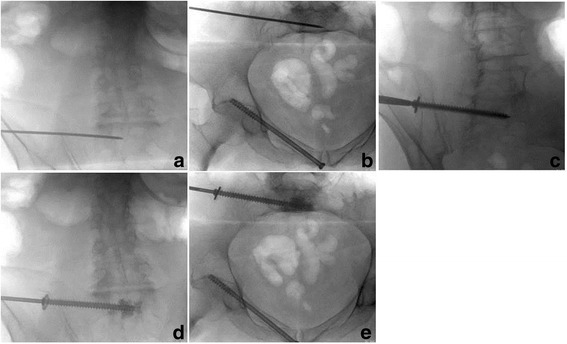
Surgical technique of in-screw cement-augmented SI screw fixation into S1 with the placement of the K-wire on the right position in inlet and outlet controls (**a** and **b**). Screw placement (**c**) and augmentation with bone-filler device in inlet and outlet views (**d** and **e**)

With the patient under anaesthesia, the insertion point was determined in lateral sacral fluoroscopic views. All screws were introduced in S1, and the correct course of the threaded K-wire was controlled with fluoroscopic inlet and outlet views [18]. The endpoint was a few millimetres over the sacral midline (Fig. 2a-b).

The screw length was measured using a length gauge over the K-wire. The K-wire was used as a guide, and a cannulated fully threaded 7.5-mm screw (Königsee Implants, Allendorf, Germany) with a washer was introduced. Afterwards, the K-wire was removed, and the bone filler system (Königsee Implants) was applied to inject PMMA (Medtronic, Santa Rosa, CA, USA) under fluoroscopic control. Generally, 3 ml of PMMA was used and spread out on the top and the perforations of the screw (Fig. 2c–e). Data of the surgical procedure including time for surgery, time to surgery, number of screws, additional anterior fixation and the used amount of cement were recorded for further analyses.

### Postoperative in-hospital stay – Postoperative pain, duration of hospital stay, adverse events

After surgery, X-rays (anterior-posterior [AP], inlet and outlet) were taken to verify the position of the implant and exclude cement leakage. CTs were indicated if postoperative neurological deficit, suspected cement leakage or thromboembolism was present.

Postoperatively patients were allowed for full weight bearing with the assistance of a physiotherapist. Supporting antiosteoporotic therapy was given or optimized for all patients according to german national guidelines [19]. Postoperative pain, duration of hospital stay, adverse events and pain using the VAS were determined.

### One-year follow-up

At the 1-year follow-up (range, 12–14 months), intensity of pain according to the VAS, mobility regarding the usage of orthopaedic aids and quality of life using the 12-Item Short Form Survey (SF-12) were investigated. Radiological examinations were performed in AP, inlet and outlet X-rays to analyse implant positioning and implant failure, which was defined as screw retraction or dislocation.

### Data analysis

All data were collected prospectively and retrospectively analysed. Data are presented as mean ± standard deviation or as medians and ranges according to the distribution type. Statistical analyses were performed using the SPSS 20.0 for Windows (SPSS, Chicago, IL, USA); Student *t*-test and Wilcoxon test were used for analysing data with Gaussian distribution and those with non-Gaussian distribution, respectively. *P* values of ≤ 0.05 were considered to be statistically significant.

## Results

### Surgical treatment

**Fig. 3:**
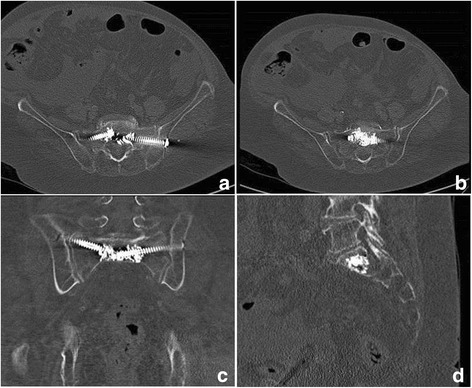
Postoperative pelvic CT of in-screw cement-augmented SI screw fixation in axial (**a** and **b**), coronal (**c**) and sagittal (**d**) views

The mean time to surgery was 4.5 ± 2.8 days after admission. In 25 cases, unilateral SI screw fixation with in-screw cement augmentation was performed in S1. In one case, fixation of two unilateral screws in S1 was performed, and in eight cases, bilateral SI screw fixation (for a total of 43 SI screws) was performed. The mean surgical time was 27 min per screw, with a range from 17 to 46 min per screw. A mean of 3.0 ± 0.6 ml of bone cement was used for augmentation (Fig. 3a–d).

Anterior stabilisation was performed either by open reduction and plate osteosynthesis (16 patients) or in cases of low displacement, by retrograde percutaneous screw fixation (16 patients). One patient received a retrograde screw on one side and plate osteosynthesis on the other side.

### In-hospital stay- hospital stay and adverse events

The mean duration of hospital stay was 14.1 ± 6.1 days (9.6 ± 6.3 days after surgical treatment). Three patients required intensive care monitoring postoperatively. In two patients, revision surgery was necessary because of the PMMA-augmented SI screw fixation. In one patient, one screw was malpositioned and perforated the ventral alar cortical border close to the iliac vessels, as shown by the inlet X-ray views and subsequent CT. In another patient, postoperative hematoma in the gluteal muscle occurred, requiring relief. Thus, complications occurred with 2 of the 43 screws implanted. In three patients, we observed non significant cement leakage into small veins (two patients) or out of the ventral sacral bone (one patient) in postoperative radiological controls, without any therapeutic consequences. No patient had neurological deficits or nerve root compression pain. Additional complications occurred in six patients; urinary tract infections in three, gastrointestinal infection with clostridia in one, pulmonary embolism in one. One patient with a Leriche syndrome not related to pelvic fixation procedure died after bypass surgery related infections 17 days after pelvic ring fracture fixation.

### Follow-up

Twenty-eight patients were available for the 1-year follow-up. Five patients were unable to completely fill out the SF-12 because of impaired mental ability. One patient refused to participate in the follow-up. Two patients were lost to follow-up. Including the patient who died during hospitalisation, four patients died within 12 months after surgery. Hence, the data of 23/34 patients were available for analyses involving the follow-up examination.

### Quality of life (SF 12) and mobility

**Fig. 4:**
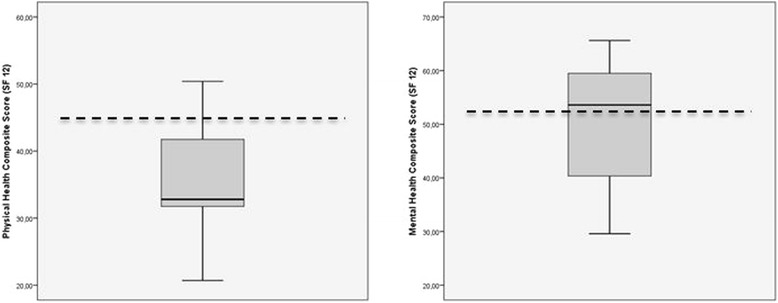
Quality of life 1 year after in-screw cement-augmented SI screw fixation according to SF-12. Dotted line shows the average for an age- and gender-matched normal German population [20]. No significantly difference was found for the physical and mental score (*p* > 0.05)

The scores for the SF-12, neither for physical and mental state, did not significantly differ from that of the corresponding age- and gender-matched population of Germany (*p* > 0.05) [20]. The average physical composite score was 35.7 ± 9.1 points, and the average mental composite score was 48.3 ± 11.8 points. These results are graphically shown in Fig. 4 [20]. Nine patients were able to walk without orthopaedic aids after 1 year, six required one or two forearm crutches and 12 were using a walker. No patient was unable to walk.

### Pain

**Fig. 5:**
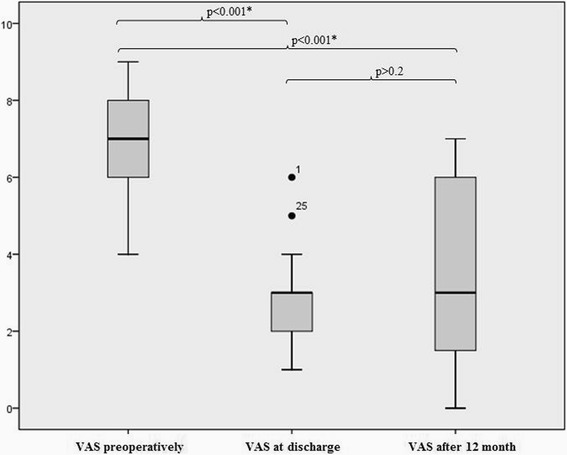
Preoperative, postoperative and 1-year pain levels according to the visual analogue pain scale

The severity of pain at the time of admission was 6.7 ± 1.4 according to the VAS. One day before discharge, the severity of pain reduced to 2.7 ± 1.0 (*p* < 0.001). The severity of pain was 3.4 ± 2.3 at the 1-year follow-up, which was not significantly different from that on the day of discharge (*p* > 0.2) (Fig. 5).

### Radiological follow-up

X-ray examination found no failures of SI screw fixation in any patient after 1 year.

## Discussion

This prospective observational study investigated the clinical safety and 1-year follow-up results of percutaneous implantation of fenestrated SI screws with in-screw PMMA augmentation. The results show that this procedure was safe and had good clinical results, including significant pain reduction, as previously described in a short-term follow-up study in elderly patients [14, 17].

Because of the increasing numbers of fragility fractures of the pelvis, several surgical procedures have been introduced to ensure early ambulation [2–4, 6, 7]. To minimise perioperative risks and comorbidities and reduce operative time in elderly patients, percutaneous techniques have been frequently used [6].

In recent years, several percutaneous techniques have been presented to treat pelvic fractures in elderly patients. For example, the use of sacroplasty led to significant pain reduction and early ambulation but had no biomechanical advantages, as shown by cyclic loadings of cadaver pelves [15, 21, 22]. Nevertheless, fracture healing appears to be impossible after PMMA is added in the fracture gap. In our opinion, the combination of osteosynthesis and a SI screw and PMMA in-screw augmentation might provide more stability and still leave the fracture zone untouched for healing.

Other approaches include the use of a trans-sacral bar, an augmented transiliacal internal fixator or a combination of sacroplasty and SI screw fixation. All techniques have resulted in significant postoperative pain reduction and early mobility; however, they have only been investigated in studies with small sample sizes [16, 23, 24]. This study has also demonstrated a significant postoperative reduction of pain, thereby supporting the results of previous studies regarding in-screw cement augmentation [14, 17].

The short surgical time of 27 min per augmented screw demonstrates that the technique is minimally invasive [25]. Moreover, the rate of complications related to the augmented screws was not higher than that for non-augmented SI screws [25, 26]. None of the complications described here were directly related to the use of PMMA and did not influence the outcome after discharge. Nonetheless, we did not use augmented screws for transforaminal sacral fractures because of a potentially higher risk for leakage of cement into the neuroforamina.

According to previously published reports, we also observed a long hospital stay of approximately 14 days and a high rate of adverse events during the hospital stay [6, 27]. The reported 1-year mortality rate after sacral fragility fractures is up to 27%, which is higher than our rate (4 of 34 patients) [28].

In our previous study we determined a significantly shorter hospital stay in the non-operative group compared to the operative group [29]. Nevertheless, approximately 20% of the non- operative group received surgery due to persistent pain and immobilization. This group most plausible corresponds to the patient population in the here presented study. In the two-year follow up there were no significant differences between all groups in pain level determined using the VAS. Moreover, as observed in the present study the physical state of all patients was lower compared to the age- and gender- adjusted German population but without achieving the level of significance. Compared to conservative treated patients also the hospital stay was shorter following conservative treatment but even shorter compared to other studies [29, 30]. Furthermore, also the complications might not differ between the different treatments and differences in deaths might be resulted from different sample sizes [29, 30].

Although these data might imply a missing superiority of the surgical treatment and especially the need for PMMA augmentation the observed higher survival following surgery might indicate a benefit by the surgical treatment [29]. Indeed, the parameters leading to this difference might be unclear and have to be elucidated. Even still alive the patient’s life might be impaired so that permanent care might be necessary as reported for conservative treatment following a pelvic ring fracture in the elderly [6]. Data for the surgical groups are still missed and should be examined in future studies.

In consideration of these data [6, 29, 30] and the excess of mortality following a pelvic ring fracture in the elderly [31], surgical treatment might be favored especially for patients suffering from persistent pain and immobilization following a conservative treatment [12].

But so far, due to osteoporosis in this patient population the rate of implant failure or backing out of the screws might be increased up to 14% as previously reported [12, 32]. Although the clinical consequence of this backing out is still unknown cement augmentation might be a promising technique to prevent this phenomenon. Thus, sacroiliac cement augmentation was introduced. Indeed, biomechanical examination failed to reveal the superiority of this technique in the hemipelvis model [33–35] the investigation of sole screw-sacral model yielded higher stability using augmented screws [36]. But so far, clinical trials using this technique especially regarding the outcome and pain are still sparse. Therefore, the present study was carried out.

A key finding is the pain reduction after 1 year with achieved mobility, which in our opinion, suggests the healing of the fracture. Without any proof, we assume reasons for the increasing distribution of the pain level after 1 year compared to discharge are co-morbidities (e.g. arthrosis, degenerative changes of the spinal column) and reduced or stopped analgesic therapy. Furthermore, the quality of life in this study population was not significantly different compared with that in the age- and gender- matched German population [20]. Moreover, in the present study no implant failure or subsequent pelvic ring fracture was observed. Regarding mobility there was no patient unable to walk suggesting also a minor amount of patients requiring permanent care.

Nonetheless, this study has some limitations. Although the study design was prospective, the rate of loss to follow-up was high. Different methods of osteosynthesis of the anterior pelvic ring might have influenced the clinical course. Because of the need for protection from radiation, not all patients underwent postoperative CT. One substantial limitation is the missed control group to compare the presented results.

## Conclusion

In-screw PMMA augmentation of fenestrated SI screws is a safe technique with a short surgical time leading to pain reduction. Follow- up data suggests non-significant differences to the age- and gender-adjusted German population and no implant failure occurred. Although the superiority of this technique compared to conservative treatment or other surgical opportunities is not examined the presented procedure might be suitable after failed conservative treatment of fragility fractures of the sacrum.

## Abbreviations

AP: anterior-posterior
CT: computer tomographie
OTA/AO: Orthopaedic Trauma Association/Association for the Study of Internal Fixation
PMMA: polymethylmethacrylate
SF-12: 12-Item Short Form Survey
SI: sacroiliac
VAS: visual analogue scale
